# A Suggestion or Aid to the Eradication of Tuberculosis by a Practical and Profitable Method

**Published:** 1895-09

**Authors:** A. S. Heath


### A SUGGESTION OR AID TO THE ERADICATION OF TUBERCULOSIS BY A PRACTICAL AND PROFITABLE METHOD.

(Continued.)

BY A. S. HEATH, M.D., V.S.

As a physiological fact and a pathological result, I assert that whatever in any degree weakens or impairs the system of man or animals renders them liable to the invasion of disease.

While the hereditary transmission of tuberculosis was maintained by the ablest pathologists of the past, the great strides of modern investigators have demonstrated that tuberculosis is a result of the invasion of a pathogenic organism, the bacillus tuberculosis, which they have shown to be infectious. While I cheerfully admit the truth of these demonstrations, and honor the genius, indomitable perseverance, and scientific skill of the able men who have demonstrated these facts, the results do not in any degree impair the truthfulness of my proposition, that whatever debilitates the constitution of man or animals renders them liable to take on disease. So far as I know, neither does Professor Koch nor his worthy co-laborers deny that a peculiar constitution renders the system susceptible to tuberculous invasion. A weak fort is easily taken. All persons exposed to infection of tuberculosis, smallpox, or scarlet fever do not take the infection, because all are not alike susceptible. Is it not from the fact that the strongest fort holds out the longest? Neither do all who eat the flesh and drink the milk of tubercular cows become tuberculous. That the system is fortified by health seems probable from the fact that physicians who are exposed to infection are less susceptible than others. That there does exist a diathesis which predisposes to tuberculosis I, for one, do not doubt. If this is so in man, why may it not also exist in animals which have also suffered from many evils of domestication? That this diathesis, this peculiar constitutional state of degeneration, is liable to hereditary transmission seems to me reasonable, since it is sustained by the views of the most renowned physicians and veterinarians, as well as by the large and long experiences of the most distinguished breeders of our domesticated animals. As in the human subject, so in in-and-inbred animals, pampered and paupered of constitution by the arts of refinement—fining the bone, thinning the skin, breeding for color or fancy, unnaturally stimulating the glandular system, overfeeding for long and injurious tests for quantity of dairy product, breeding together animals thus debilitated year in and year out—has brought upon some of our herds a susceptibility to tuberculosis that imminently threatens the public health.

Such a condition in our dairy herds is as expressive to the physician of comparative medicine as the following characteristic diathesis must be to the human practitioner: A clear complexion, a fine skin, beautiful and finely-cut features, roseate tint of lip, pearly white teeth, large and lustrous eyes, widely dilated pupils, and the white of the eye as brilliant as the pearly teeth. Let us go a little further, though, perhaps, not necessary. Long and curved eyelashes of silky texture, deep blue veins showing through the translucent skin, the light fine bones as shown in the small hands and feet, the tall, thin, and graceful body and the fine nervous organization and the brilliant intellectual faculties, all proclaiming a painful lack of stamina, strength, and longevity. Similar characteristics relatively in our domesticated animals must certainly typify a lack of constitution, vigor, and prepotency not sought for by the judicious breeder.

That the preservation of his health and the attainment of longevity are the most ardent desires of every man of sane mind is undoubted. It is also true that the wise breeder of domesticated animals breeds for perpetuity of excellence and for profit. These can only be secured by breeding from sound, healthy animals and for the best products.

Breeding animals must be healthy—free from defects of form and constitution, from tendency to weakness or disease, from ill temper or bad habits; must have sound digestive organs, capable of prompt digestion, and quick and perfect assimilation of food. The breeder must intimately know the capabilities and characteristics of his breeding animals, so as to be able to adapt them to rear young which shall answer his preconceived wants. Though food, climate, soil, altitude, exposure, shelter, care, kindness, and many other operating circumstances may all produce great changes, yet, all operating at the same time and for a long period on the animal and its progeny, cannot change the species, though it may secure many desirable excellences.

By selection, we can in time breed small-boned animals into large-boned ones, long-legged ones into short-legged ones; we can breed the horned into hornless ones. In a word, by selection the breeder can make the black white, the white black, the barren fruitful, the deformed straight, the imperfect perfect; he can breed to a feather; he can produce a tendency to meat, to milk, to butter, to cheese, to capacity to labor, for speed, for endurance, or to serve almost any reasonable desire, demand, or fancy. By breeding from carefully-selected parents he can rapidly increase his flocks and herds by choosing those of great fecundity from which to breed—ewes from families that give twins, from cows that uniformly breed, from sows that farrow large numbers of pigs—and it is just as essential that the males, also, should be selected from like prolific families of sires and dams. It is in this potential selection that the hope of breeding out the tendency to tuberculosis becomes a factor of vast possibilities.

The Norman breed of cattle, free from tuberculosis and its tendency, with a constitution of iron, and with the largest capabilities for beef and for the most valuable dairy products, with perfect health, prepotency, size, and constitution, must prove a valuable factor in the eradication of tuberculosis from our dairy herds.

If we could breed strong, healthy men as we can breed animals with like characteristics, the fearful scourge of tuberculosis would be banished from our race.

For the commercial value of my proposition I must defer for another communication.

